# Discovery of Biomarkers for Amyotrophic Lateral Sclerosis from Human Cerebrospinal Fluid Using Mass-Spectrometry-Based Proteomics

**DOI:** 10.3390/biomedicines11051250

**Published:** 2023-04-23

**Authors:** Sungtaek Oh, Yura Jang, Chan Hyun Na

**Affiliations:** 1 Department of Neurology, Institute for Cell Engineering, Johns Hopkins University School of Medicine, 733 N. Broadway, Baltimore, MD 21205, USA; 2 Adrienne Helis Malvin Medical Research Foundation, New Orleans, LA 70170, USA

**Keywords:** amyotrophic lateral sclerosis, cerebrospinal fluid, proteomics, mass spectrometry, biomarker

## Abstract

Amyotrophic lateral sclerosis (ALS) is a progressive neurodegenerative disease characterized by the loss of upper and lower motor neurons, which eventually may lead to death. Critical to the mission of developing effective therapies for ALS is the discovery of biomarkers that can illuminate mechanisms of neurodegeneration and have diagnostic, prognostic, or pharmacodynamic value. Here, we merged unbiased discovery-based approaches and targeted quantitative comparative analyses to identify proteins that are altered in cerebrospinal fluid (CSF) from patients with ALS. Mass spectrometry (MS)-based proteomic approaches employing tandem mass tag (TMT) quantification methods from 40 CSF samples comprising 20 patients with ALS and 20 healthy control (HC) individuals identified 53 proteins that are differential between the two groups after CSF fractionation. Notably, these proteins included both previously identified ones, validating our approach, and novel ones that have the potential for expanding biomarker repertoire. The identified proteins were subsequently examined using parallel reaction monitoring (PRM) MS methods on 61 unfractionated CSF samples comprising 30 patients with ALS and 31 HC individuals. Fifteen proteins (APOB, APP, CAMK2A, CHI3L1, CHIT1, CLSTN3, ERAP2, FSTL4, GPNMB, JCHAIN, L1CAM, NPTX2, SERPINA1, SERPINA3, and UCHL1) showed significant differences between ALS and the control. Taken together, this study identified multiple novel proteins that are altered in ALS, providing the foundation for developing new biomarkers for ALS.

## 1. Introduction

Amyotrophic lateral sclerosis (ALS) is a progressive neurodegenerative disease characterized by the loss of upper and lower motor neurons, eventually leading to death [1,2,3,4,5]. An average of 5.5 people per 100,000 population are annually diagnosed with ALS in the United States [6,7,8]. ALS patients die within 2–5 years after the first diagnosis [1,9]. Clinical hallmarks of ALS have significant heterogeneity, including behavioral changes for ≈60% of the cases and co-occurrence of frontotemporal dementia for ≈15% of the cases [10]. The etiology of ALS is still elusive. Although 5–10% of ALS cases are familial, most cases are sporadic [11]. The commonly known genetic risk factors of ALS include changes in *C9ORF72*, *SOD1*, *TARDBP*, and *FUS* [9]. Genetic changes in *C9ORF72*, which is the most frequently observed in ALS, are autosomal dominant and manifested by several hundred to thousands of hexanucleotide repeats of GGGGCC in the promotor or intron1 of the gene [12]. More than 170 genetic alterations are observed in *SOD1* in ALS, and these are also autosomal dominant. Because most of the mutations in *SOD1* are observed in exons, it is widely accepted that SOD1 protein dysfunction is linked to ALS pathogenesis [11,13]. *TARDBP* encodes TDP43, a DNA- and RNA-binding protein involved in the splicing, transcription, stability, and transport of mRNAs. TDP-43 is implicated in ALS through gain-of-function mechanisms in which mutant TDP-43 disrupts stress granules or via loss-of-function in which TDP-43 is depleted from the nucleus [11,14]. *FUS* encodes a nucleoprotein that colocalizes with TDP-43 and is considered to have similar RNA editing deficiencies as TDP-43 in ALS [15].

Since there is no cure for ALS, current treatment options are focused on slowing progression and managing symptoms. For instance, riluzole is employed to inhibit glutamate release from pre-synaptic terminals, while edaravone acts as a free radical scavenger, thus slowing the disease’s progression [16]. On the other hand, baclofen is utilized to alleviate spasticity, while nuedexta is utilized to alleviate both spasticity and difficulties with swallowing [16,17]. Therefore, the development of biomarkers that can be used for elucidating disease mechanisms and for the diagnosis, prognosis, and estimation of pharmacodynamics is imperative. Recently, there have been major efforts dedicated to identifying biomarkers for ALS, and chief among these are quantitative methods to measure proteins in patient biofluids such as blood, urine, saliva, and cerebrospinal fluid (CSF). Mass spectrometry (MS)-based proteomic approaches are considered the gold standard for protein discovery, and there have been multiple attempts to develop protein biomarkers for ALS using proteomics technology [5,6,18,19,20,21,22]. So far, multiple proteins, including CHIT1, CHI3L1, CHI3L2, UCHL1, MAP2, GPNMB, and neurofilament proteins, have been reported as candidate ALS biomarkers [23,24,25,26]. Many of these proteins await rigorous validation [27]. Clearly, the discovery of additional novel biomarkers is needed, but one major barrier is that many key regulatory proteins are of relatively low abundance and fail to be detected [28]. Accordingly, the identification of useful biomarkers for ALS requires the application of innovative, highly sensitive, and accurate methodologies. Herein, we report findings from an initial unbiased proteomics discovery study on CSF from 20 patients with ALS and 20 healthy controls (HC). We employed tandem-mass-tag (TMT)-based multiplexing technology for the accurate and sensitive quantification of CSF proteins, with subsequent analysis of TMT-labeled peptides via an Orbitrap Fusion Lumos ETD MS. From the discovery phase, we identified several new candidate ALS biomarker proteins as well as several previously reported ones, validating our approach. Using proteins identified from the discovery phase, we subsequently performed a validation experiment using parallel reaction monitoring (PRM) targeted MS in 61 CSF samples from 30 patients with ALS and 31 HC individuals. Through these approaches, we identified novel biomarkers for ALS that can be used for the diagnosis, prognosis, and estimation of pharmacodynamics, which will contribute to a better understanding of ALS pathogenesis mechanisms. Further, this work highlights that our innovative MS approach has iterative potential in other neurodegenerative diseases.

## 2. Methods

### 2.1. Collection of CSF Samples

CSF samples from 11 HC individuals and 30 patients with ALS were obtained from the Northeast Amyotrophic Lateral Sclerosis (NEALS) consortium. Nineteen additional CSF samples from HC individuals were provided by the Advancing Research and Treatment for Frontotemporal Lobar Degeneration Research Consortium (ARTFL) at the University of California San Francisco (UCSF). Lumbar punctures were performed using the atraumatic technique and collected in a polypropylene tube before transferring to a 50 mL conical polypropylene tube at room temperature (RT), which was mixed gently by inverting 3–4 times. Within 15 min of collection, CSF was centrifuged at 2000× *g* for 10 min at RT and aliquoted directly into pre-cooled polypropylene cryovials. Within 60 min of CSF collection, aliquots were frozen on dry ice and then stored at −80 °C, until further analysis. For samples collected prior to 2015, the protocol used is described by Scherling CS et al. [29]. Study participants provided written informed consent, and all procedures were approved by the relevant Institutional Review Boards (IRB). The demographics of CSF samples used in this study are provided in Table 1.

### 2.2. Sample Preparation and Trypsin Digestion for Discovery Experiments

**Figure 1:**
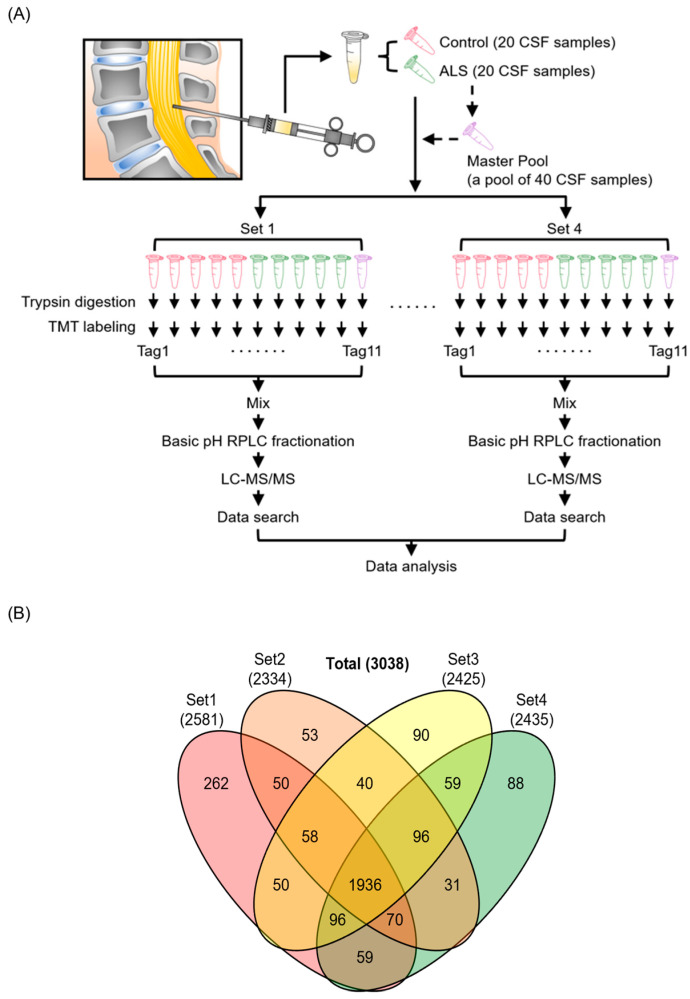
Experimental strategy and identified proteins from the discovery experiment for ALS biomarker candidate proteins. Human CSF samples from 20 patients with ALS and 20 HC individuals were analyzed with four 11-plex TMT experimental batches. MP was prepared by pooling all 40 CSF samples and was added to each TMT experiment to normalize between batches. CSF proteins were digested with trypsin and labeled with TMT reagents, followed by pre-fractionation into 24 fractions prior to mass spectrometry analysis. Proteins were subsequently identified by conducting a database search of the acquired mass spectra (**A**). The numbers of identified proteins from 4 batches of TMT-based proteomics experiments are shown in the Venn diagram (**B**).

Sample preparation was conducted as described previously with minor modifications [30,31]. To analyze 40 CSF samples using an 11-plex TMT, the 40 CSF samples were divided into 4 batches of 10 samples each, and a master pool (MP) sample was included in each batch for normalization between 4 batches. The MP was prepared by combining an equal volume from all 40 CSF samples including HC and ALS (Figure 1A). To denature proteins in CSF, we mixed each CSF sample with one volume of lysis buffer composed of 10 M urea, 100 mM triethylammonium bicarbonate (TEAB), 20 mM tris (2-carboxyethyl) phosphine hydrochloride (TCEP), and 80 mM chloroacetamide (CAA), followed by incubating the samples at RT for 1 h for reduction and alkylation to be completed. Subsequently, the protein digestion was carried out with LysC (lysyl endopeptidase MS grade, Fujifilm Wako Pure Chemical Industries Co., Ltd., Osaka, Japan) at a one-to-fifty ratio for 3 h at 37 °C, followed by trypsin (sequencing grade modified trypsin, Promega, Fitchburg, WI, USA) digestion overnight at 37 °C after diluting urea from 5 M to 2 M with 50 mM TEAB. After acidifying digested peptides with 1% trifluoroacetic acid (TFA) to the final concentration, they were desalted using C_18_ StageTips (3M Empore™, St. Paul, MN, USA). The desalted peptides were dried with a Savant SPD121P SpeedVac concentrator (Thermo Fisher Scientific, Waltham, MA, USA). To perform TMT-based quantitative MS, the digested peptides from CSF samples were labeled using 11-plex TMT reagents following the manufacturer’s instructions (Thermo Fisher Scientific). The MP samples were labeled with 131C, while CSFs from ALS patients and HC individuals were labeled with the rest of the TMT tags at RT for 1 h. Labeling reactions were carried out at RT for 1 h after mixing each peptide sample in 100 mM TEAB with TMT reagent in acetonitrile (ACN). After this, the reaction was quenched by adding 1/10 volume of 1 M Tris-HCl (pH 8.0). The TMT-labeled peptides were pooled and pre-fractionated by basic pH reversed-phase liquid chromatography (bRPLC) with an Agilent 1260 offline HPLC system (Agilent Technologies, Santa Clara, CA, USA) as follows. The peptides were reconstituted in solvent A (10 mM TEAB in water, pH 8.5); loaded onto an Agilent 300 Extend-C_18_ column (5 m, 4.6 mm × 250 mm, Agilent Technologies); and resolved over 90 min of a gradient of solvent B (10 mM TEAB in 90% ACN and 10% water, pH 8.5) at 0.3 mL/min, collecting 96 fractions. After this, the 96 fractions were concatenated into 24 fractions and dried in a SpeedVac.

### 2.3. LC-MS/MS Analysis for the Discovery of Biomarker Candidates

LC-MS/MS analysis was conducted as described previously with minor modifications [30,31]. The dried peptides were reconstituted in 0.5% formic acid (FA) and analyzed using an Orbitrap Fusion Lumos Tribrid MS coupled with an Ultimate 3000 RS Autosampler nanoflow liquid chromatography system (Thermo Fisher Scientific). The peptides were loaded into trap columns (Acclaim™ PepMap™ 100 LC C_18_, 5 μm, 100 μm × 2 cm, Thermo Fisher Scientific) at a flow rate of 8 μL/min and separated on an analytical column (Easy-Spray™ PepMap™ RSLC C_18_, 2 μm, 75 μm × 50 cm, Thermo Fisher Scientific) at a flow rate of 0.3 μL/min with mobile phases consisting of 0.1% FA in water and 0.1% FA in 95% ACN and 5% water. The overall run time was 120 min. The MS data were acquired in a data-dependent acquisition mode. The MS1 scan range for precursor ions was set to *m*/*z* 300–1800 in the top-speed mode. The resolutions for MS1 and MS2 were set to 120,000 and 50,000, respectively, at an *m*/*z* of 200. The automatic gain controls for MS1 and MS2 were set to 1 × 10^6^ and 5 × 10^4^, respectively. The maximum ion injection times for MS1 and MS2 were set to 50 ms and 100 ms, respectively. The charges for MS2 isolation were set to 2 to 5, and the most intense ions were fragmented using higher-energy collisional dissociation (HCD) fragmentation with 35% normalized collision energy. The precursor isolation window was set to *m*/*z* 1.6 with a *m*/*z* 0.4 offset and the dynamic exclusion was set to 30 s. The internal calibration was performed using the lock mass option (*m*/*z* 445.1200025) from ambient air.

### 2.4. Database Searches for Peptide and Protein Identification

The database search and quantification were conducted as described previously with minor modifications [30,31]. A database search of the acquired tandem MS data was conducted against the human UniProt database (released in May 2018, containing protein entries with common contaminants) with the SEQUEST search algorithm embedded in the Thermo Proteome Discoverer platform (version 2.2.0.388, Thermo Fisher Scientific). The top ten peaks within each *m*/*z* 100 window were selected for database searches during MS/MS preprocessing. The database search parameters were as follows. Two missed cleavages by trypsin were allowed. Carbamidomethylation (+57.02146 Da) at cysteine and TMT modification (+229.162932 Da) at the N-terminus of peptides and lysine residues were set for fixed modifications, while oxidation (+15.99492 Da) of methionine was set for variable modifications. The MS1 error tolerance was set to 10 ppm, and the MS/MS error tolerance was set to 0.02 Da. The minimum peptide length was set to 6 amino acids. False discovery rates (FDRs) were set at 1% for both peptides and proteins. The protein quantification was performed with the parameters as follows. A centroid-based integration mode was used, a tolerance of 20 ppm was set for reporter ions, MS order was set to MS2, and the activation type was set to HCD. The quantification value correction was disabled. Peptide quantification was performed using both unique and razor peptides. Peptide uniqueness was determined at the level of protein groups. Missing intensity values were replaced with the minimum value. The signal-to-noise ratio was used to compute reporter ion abundance. The quantification value corrections for isobaric tags were disabled. A threshold of 50% was set for co-isolation. The average signal-to-noise threshold for reporters was set to 50. Data normalization was disabled. Protein groups were created by applying the strict parsimony principle as follows: (1) all proteins that share the same set or subset of identified peptides were grouped; (2) protein groups without any unique peptides were filtered out; and (3) Proteome Discoverer iterated through all spectra and chose which peptide-spectrum match (PSM) to use in ambiguous cases to create a protein group with the greatest number of unambiguous and unique peptides. The Proteome Discoverer summed all the reporter ion abundances of PSMs for the corresponding proteins in a TMT run.

### 2.5. Preparation of CSF Samples for the PRM Experiments

The isotopically labeled peptides (SpikeTides L, JPT Peptide Technologies GmbH, Berlin, Germany) were synthesized with ^13^C- and ^15^N-labeled lysine and arginine at their C-termini and were used for the PRM analysis. The CSF samples were mixed with 1 volume of the lysis buffer, composed of 8 M urea, 20 mM TCEP, 80 mM CAA, 100 mM TEAB, and synthetic peptides with known amounts. The CSF and lysis buffer mixture were incubated for 1 h at RT for reduction and alkylation. Protein digestion was carried out using LysC (Wako Chemicals Industries Co., Ltd., Osaka, Japan) at a one-to-fifty ratio at 37 °C for 3 h and then using trypsin (sequencing grade modified trypsin, Promega, Fitchburg, WI, USA) at a one-to-fifty ratio at 37 °C overnight after diluting the concentration of urea from 4 M to 2 M by adding 50 mM TEAB. Peptides were purified using C_18_ StageTips (3M Empore™, St. Paul, MN, USA) after acidifying with TFA. The peptide eluents were dried in a SpeedVac (Thermo Fisher Scientific) and then stored at −80 °C until use.

### 2.6. LC-MS/MS Analysis for Validation Experiments Using the PRM Method

LC-MS/MS analyses were conducted as described previously with minor modifications [30,31]. The peptide samples were analyzed on an Orbitrap Fusion Lumos Tribrid mass spectrometer interfaced with an Ultimate 3000 RSLCnano nanoflow liquid chromatography system (Thermo Fisher Scientific). The peptides reconstituted in 15 μL of 0.1% FA were loaded on Acclaim PepMap100 Nano-Trap column (100 μm × 2 cm, Thermo Fisher Scientific) packed with 5 μm C_18_ particles at a flow rate of 5 μL per min. The flow rate employed was 250 nL/min using a linear gradient of 6% to 28% solvent B (0.1% FA in 95% ACN and 5% water) over 55 min on an EASY-Spray column (50 cm × 75 µm, Thermo Fisher Scientific) packed with 2 µm C_18_ particles (Thermo Fisher Scientific), which was fitted with an EASY-Spray ion source operated at a voltage of 2.0 kV. Mass spectrometry analysis was conducted in the targeted MS2 mode. The MS1 scan range for a full survey scan was acquired from *m*/*z* 300 to 1600 with a resolution of 120,000 at an *m*/*z* of 200. The mass resolution for MS2 was set to 30,000 at an *m*/*z* of 200. Automatic gain control was set to 500,000 and 100,000 ions for MS1 and MS2, respectively. The maximum ion injection time and HCD fragmentation energy for each peptide are listed in Table S1.

### 2.7. Experimental Design and Statistical Rationale

The sample size determination was conducted in a similar way to the one described previously with minor modifications [30,31]. The total number of CSF samples used in the discovery study was 40, composed of 20 ALS patients and 20 HC individuals. We conducted sample size analysis using the pwr package in R. When we wanted to detect proteins with 1.5-fold differences between groups, the required minimum sample size was 11.299 when the significance level was 0.0001, power was 0.8, sigma was 0.24, and delta was 0.585 (=log_2_1.5). This sigma value of 0.24 was derived from our in-house TMT proteomics experiments conducted with human CSF samples. The significance level of 0.0001 was determined on the basis of our in-house data. When we identified several thousands of proteins, the majority of the proteins with *p* value < 0.0001 showed *q*-value < 0.05. On the basis of this sample size analysis, we decided to use 20 samples per group. The statistical analysis of the mass spectrometry data was performed with the Perseus (version 1.6.0.7) software package. The intensity values of each protein were divided by the one of the MP included in each batch, followed by dividing the values of each sample by the sample median. The relative abundance values for each sample were log_2_-transformed, followed by *z*-score transformation [32,33]. The fold changes between the comparison groups were calculated by dividing the average abundance values of each protein from one group by the values from another group. According to our normality test using the Shapiro–Wilk test in the dplyr package in R, the majority of the proteins showed normal distribution. Thus, *p* values between the comparison groups were calculated by Student’s two-sample *t*-test. Since we were conducting multiple comparisons, we calculated an FDR by comparing data with and without permutations between groups. The *q*-values for the volcano plots were calculated by a permutation-based FDR estimation in the significance analysis of microarrays (SAM) method, in which *p* values and fold-changes were calculated before and after the permutation of samples from two groups [34]. Protein–protein association network analysis was conducted using the STRING database [35].

Skyline software (version 21.2) was used for the analysis of PRM data [36]. The quantification was conducted by summing the area under the curves (AUC) of the third-to-last fragment *y* ions of the selected peptides. Statistical analysis was performed using GraphPad Prism (version 9, GraphPad Software Inc., San Diego, CA, USA). Student’s unpaired, two-tailed *t*-test was used for the comparison between HC with ALS. A probability value of *p* < 0.05 was considered statistically significant. To evaluate the performance of biomarker candidates, univariate and multivariate receiver operating characteristic (ROC) curve analysis was performed in Metaboanalyst (version 5.0) [37]. For the univariate ROC analysis, bootstrapping was conducted by resampling 500 times to generate an AUC mean value with a 95% confidence interval. For multivariate ROC analysis, the partial least squares discriminant analysis (PLS-DA) classification method with the feature ranking method built in PLS-DA was selected. The number of latent variables was set to 2. To begin multivariate analysis using PLS-DA, ROC curves were generated using balanced subsampling by Monte Carlo cross-validation (MCCV). In each MCCV, 2/3 of the samples were used to evaluate feature importance, and the remaining 1/3 were used to validate the models created with the first step. The top-ranking features in terms of importance were used to build the biomarker classification models. This was repeated several times to calculate the performance and confidence intervals of each model.

### 2.8. Disclosure of Previously Published Data

Out of ≈3000 proteins identified in the TMT-based discovery experiment of this study, the data for 78 glycosylphosphatidylinositol-anchored proteins (GPI-APs) were published previously to show the differential release of GPI-APs by differential glycerophosphodiester phosphodiesterase 2 activity in ALS patients compared to control individuals [38].

## 3. Results

### 3.1. Quantitative Proteome Analysis of CSF Samples

We first implemented an unbiased discovery-based approach to identify proteins that are differentially expressed in the CSF of patients with ALS compared with HC individuals. We performed a quantitative proteome analysis of 40 CSF samples comprising 20 patients with ALS and 20 HC individuals using TMT labeling-based mass spectrometry (Figure 1A, Table 1). The 40 samples were split into 4 batches of 10 and were labeled with an 11-plex TMT reagent. The MP, which is a reference sample prepared by pooling equal volumes of all 40 CSF samples, was placed at the 11th channel of each 11-plex TMT experimental set for the purpose of normalization between batches. After enzyme digestion and TMT labeling, the peptides were pre-fractionated using a bRPLC system. The fractions were analyzed by LC-MS/MS. In total, 3,828,053 MS/MS spectra were acquired, and 402,387 MS/MS spectra were assigned to peptides leading to the identification of 26,726 peptides and 3038 proteins. Out of 3038 proteins, 1936 proteins were identified in all four TMT experimental batches (Figure 1B, Supplementary Data S1).

### 3.2. Selection of Candidate Biomarkers for ALS

**Figure 2:**
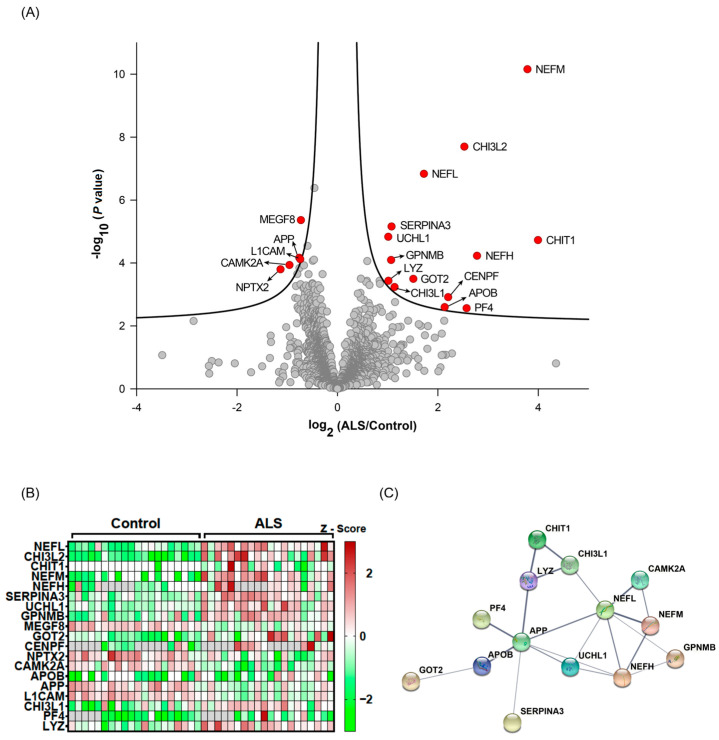
Statistical and bioinformatic analyses of the identified CSF proteins. The quantified CSF proteins from 20 patients with ALS and 20 HC individuals were plotted on a volcano plot. The curved lines are the boundaries for a *q*-value of 0.05. The proteins with *q*-value < 0.05 are colored in red (**A**). The differentially expressed proteins (*q*-value < 0.05) are shown on a heatmap (**B**). Protein–protein association network analysis (**C**) for differentially expressed proteins (*q*-value < 0.05) using STRING.

To quantify protein abundances measured by the four TMT experimental batches, we normalized the intensity values of each protein to the intensities of the MP samples in each TMT experiment. The normalized data were subsequently subjected to statistical analyses to identify proteins that were differentially expressed between the two groups. Nineteen proteins were found to have differential expression between ALS and HC on the basis of *q*-value < 0.05 (Table 2, Figure 2A,B). Out of 19 differentially expressed proteins, 15 proteins were upregulated in ALS, while only 4 proteins were downregulated in ALS. Twelve proteins (SERPINA3, GPNMB, MEGF8, GOT2, CENPF, NPTX2, CAMK2A, APP, L1CAM, CHI3L1, PF4, and LYZ) were novel, and seven proteins (CHI3L2, CHIT1, NEFL, NEFM, NEFH, UCHL1, and APOB) were reported previously [23,24,25,26,39,40]. Protein–protein association network analysis showed that NEFL and APP were the most connected proteins within the panel of proteins identified (Figure 2C). Taken together, this discovery-based approach successfully identified potential biomarkers for ALS.

### 3.3. Detectability of Candidate Biomarker Proteins in Targeted PRM Analysis

To validate the potential of the proteins identified in the discovery experiments as biomarkers for ALS, we opted to use PRM analysis of unfractionated CSF. PRM is a targeted mass spectrometry analysis that does not utilize the chemical modification of peptides. Instead, known quantities of standard isotope labeled (SIL) tryptic peptides that have identical sequences to the tryptic peptides of the target proteins were spiked into the sample for accurate quantitation of the relevant endogenous target peptides. We first determined whether peptides of the target proteins were detectable by PRM analysis in unfractionated CSF samples. CSF samples were pooled from 20 patients with ALS and 20 HC individuals, and 200 fmol of SIL peptides corresponding to the endogenous target peptides were added prior to trypsin digestion to enable subsequent monitoring of relevant peptides. We evaluated 53 proteins with *q*-value < 0.1 (Table 2), and 31 of the 53 proteins were detectable by PRM (Table 2). A total of 15 of the 31 proteins were detected by two or more peptides, while 18 proteins were detected by one peptide (Table S1).

### 3.4. Response Curve Test of Candidate Biomarker Proteins in PRM Analysis

Prior to beginning the PRM analysis, we examined the response curve of the target peptides to determine the proper range for accurate quantification of the 16 target proteins. We added varying amounts of SIL peptides (0.01, 0.1, 1, 10, 100, 1000, and 10,000 fmol) corresponding to the target tryptic peptides from the pooled CSF samples, and the SIL peptides were quantified by PRM analysis. The majority of the peptides showed linear responses in the range from 0.1 fmol to 10 pmol (Figure S1). Five peptides, namely, APOB (WNFYYSPQSSPDK), CAMK2A (FYFENLWSR), CAMK2A (ITQYLDAGGIPR), CAMK2A (VTEQLIEAISNGDFESYTK), and CHIT1 (DNQWVGFDDVESFK), showed linear responses at the range from 0.01 fmol to 10 pmol (Figure S1A). Twenty-nine peptides, namely, IGLV3-19 (ITCQGDSLR), JCHAIN (FVYHLSDLCK), LTA4H (WEDAIPLALK), NFASC (LTVSWLK), PPBP (ICLDPDAPR), UCHL1 (FSAVALCK), CHIT1 (YPLIQTLR), ERAP2 (ILAVTDFEPTQAR), FSTL4 (FDDYNSDSSLTLR), APP (THPHFVIPYR), APP (VESLEQEAANER), APP (WYFDVTEGK), CHI3L1 (ILGQQVPYATK), CNTNAP2 (FSFSTTK), MEGF8 (LFPLPGR), MFAP4 (GFYYSLK), NELL2 (SALAYVDGK), NPTX2 (AAVLQLR), NPTX2 (TESTLNALLQR), RTN4RL2 (LFLQNNLIR), SERPINA3 (LINDYVK), CHI3L1 (QLLLSAALSAGK), NPTX1 (LENLEQYSR), NPTX1 (LPFVINDGK), RTN4RL2 (SLEPDTFQGLER), SERPINA1 (LSSWVLLMK), SERPINA1 (SVLGQLGITK), TPI1 (QSLGELIGTLNAAK), and MFAP4 (ADGEYWLGLQNMHLLTLK), showed linear responses at the range from 0.1 fmol to 10 pmol (Figure S1B–D). Sixteen peptides, namely, GPNMB (AYVPIAQVK), JCHAIN (IVLVDNK), L1CAM (YDIEFEDK), ST8SIA5 (EINSADFVFR), APOB (LPYTIITTPPLK), CHI3L1 (FPLTNAIK), CLSTN1 (YISNEFK), CSPG5 (EAGSAVEAEELVK), LINGO1 (HLNINAIR), MEGF8 (HVWTTLK), NCAN (ELGGEVFYVGPAR), NCAN (LSSAIIAAPR), NCAN (QDLPILVAK), NELL2 (AFLFQDTPR), NELL2 (ASTATAEQFFQK), and NELL2 (FTGSSWIK), showed linear responses at the range from 1 fmol to 10 pmol (Figure S1E,F). Four peptides, namely, CHIT1 (SSFYSCAAGR), CHIT1 (FTDMVATANNR), CLSTN3 (FTVTAYDCGK), and FSTL4 (LLVESLFR), showed linear responses at the range from 10 fmol to 10 pmol (Figure S1G). Two peptides, namely, SERPINA3 (AVLDVFEEGTEASAATAVK) and SERPINA1 (LYHSEAFTVNFGDTEEAK), showed linear responses at the range from 100 fmol to 10 pmol (Figure S1G). In summary, almost all target peptides showed a linear response curve in the detectable range, enabling us to quantify target peptides accurately.

### 3.5. Targeted Quantification of Candidate ALS Biomarker Peptides in CSF from ALS and HC Individuals

**Figure 3:**
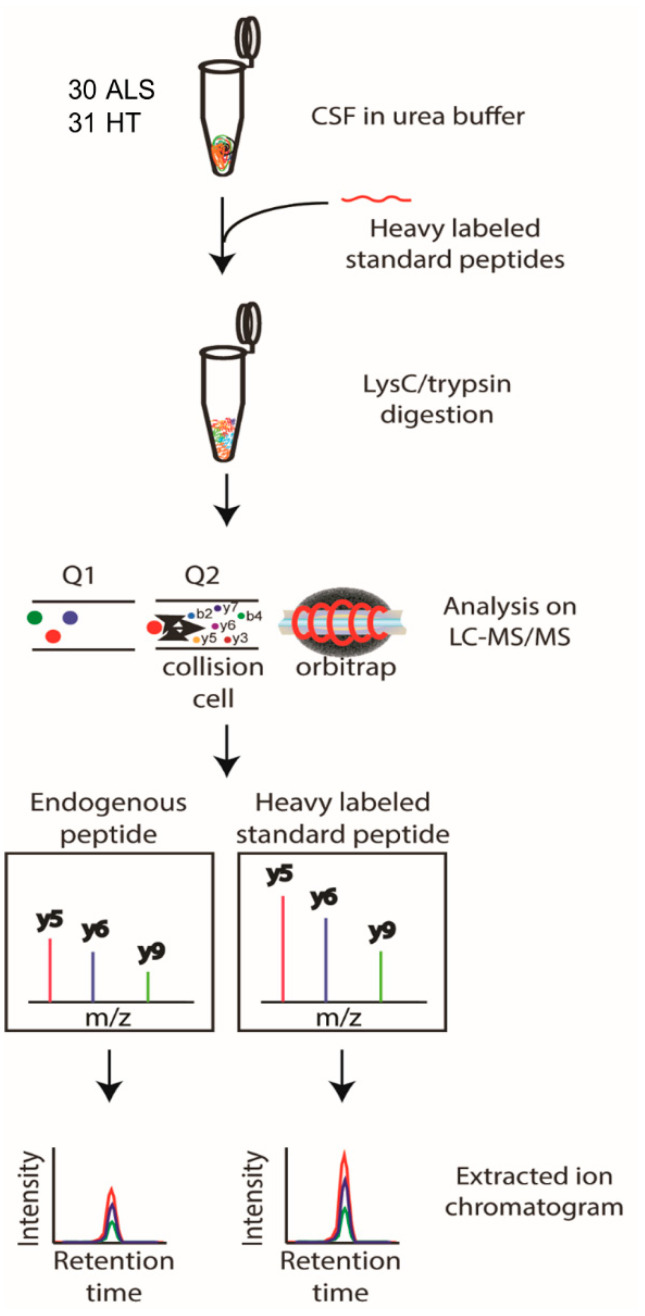
Schematic workflow of the PRM analysis. The selected candidate ALS biomarker proteins were validated by PRM analysis of CSF samples from 30 ALS patients and 31 HC individuals. SIL peptides were added to the CSF samples for the accurate quantification of the target peptides, followed by trypsin digestion and PRM analysis. The peptide abundance was calculated by extracting the AUC of light endogenous and heavy SIL peptides.

**Figure 4:**
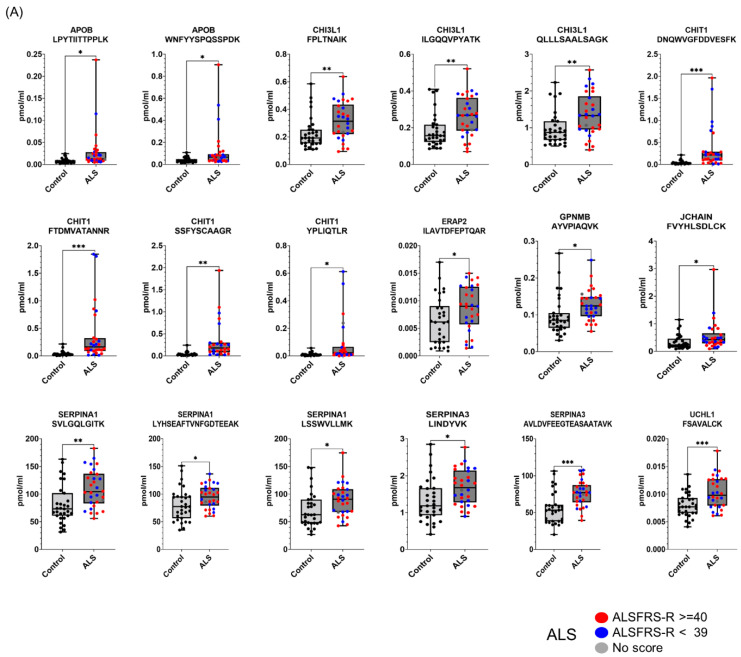

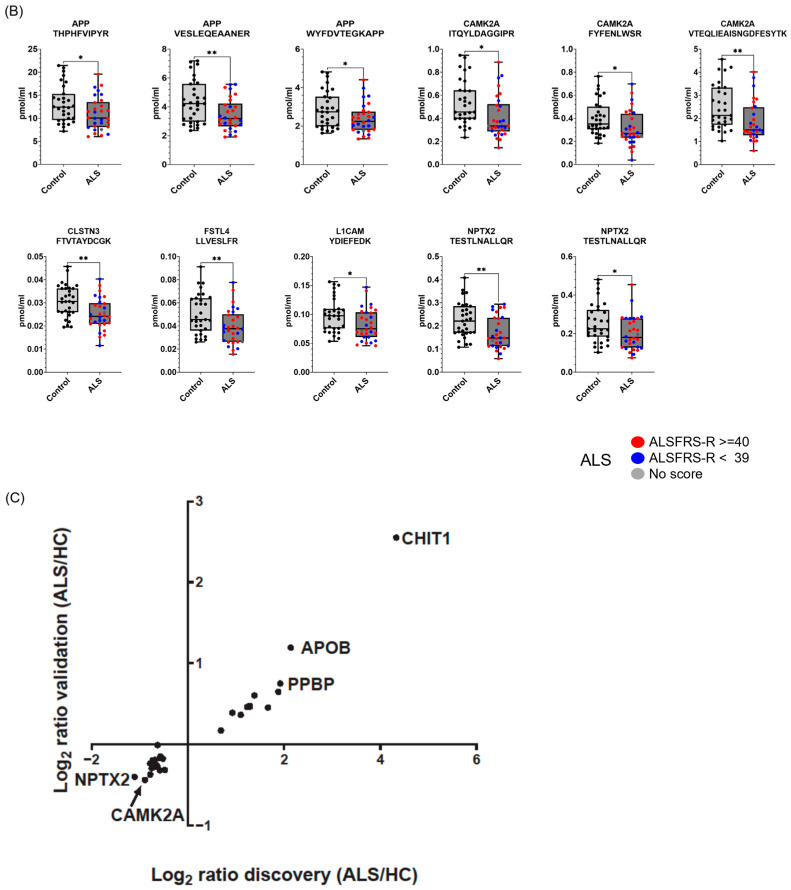
Proteins differentially expressed in ALS CSFs in the validation experiment. The selected candidate ALS biomarker proteins were validated by PRM analysis using 30 ALS patients and 31 HC individuals. SIL peptides were added to the CSF samples for the accurate quantification of the target peptides for the PRM analysis. The peptide abundance was calculated by extracting the AUC of light endogenous and heavy SIL peptides. Eighteen peptides were upregulated in ALS (**A**), and eleven peptides were downregulated in ALS (**B**). Patients with ALSFRS scores < 39 are shown as blue dots, and patients with ALSFRS scores ≥ 40 are shown as red dots. Since one ALS patient had no score, the patient is shown as a gray dot. All PRM-MS analyses were performed in 3 technical replicates. Student’s unpaired, two-tailed *t*-test was conducted for statistical analysis between groups (* *p* < 0.05; ** *p* < 0.01; *** *p* < 0.0001). The correlation of candidate biomarkers measured in the discovery and validation experiment is shown (**C**).

**Figure 5:**
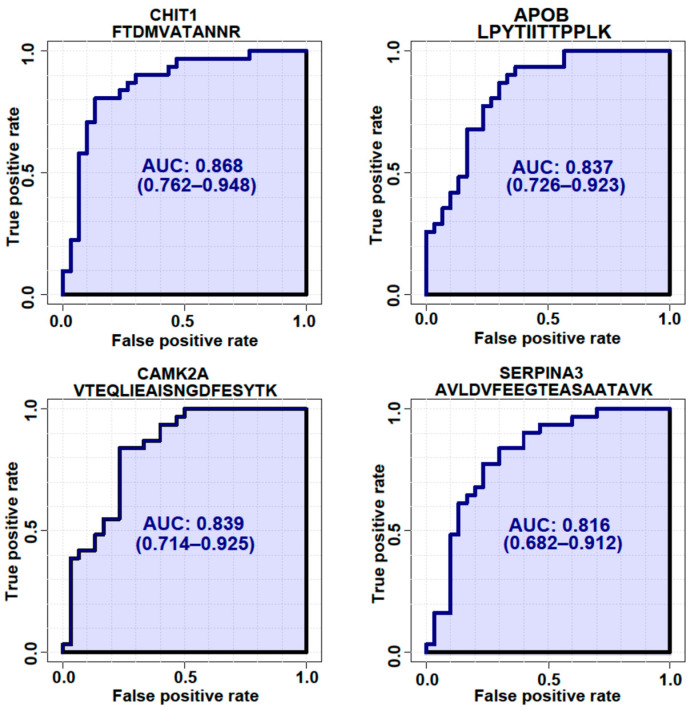
ROC analysis of 4 representative proteins with the highest AUCs. ROC analysis was generated by bootstrapping. The values in parentheses show the lower and upper AUC values of the 95% confidence interval.

**Figure 6:**
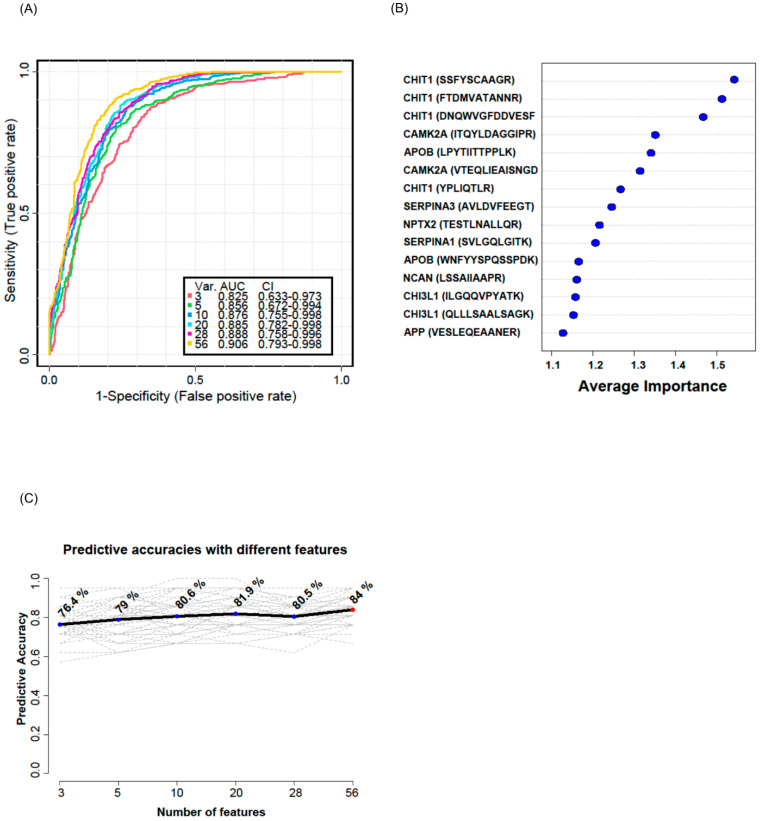
Multivariate ROC analysis and predictive accuracy. Multivariate ROC analyses were conducted using different numbers of features ranging from 3 to 56. Var. indicates the number of features used. CI indicates confidence interval (**A**). The top 15 significant features affecting the discrimination of ALS from HC are shown with their average importance (**B**). The accuracy for predicting ALS as the number of features increased is shown (**C**).

To validate whether the selected candidate peptides show differential expression levels in CSF from ALS patients without TMT labeling, we quantified the target peptides using PRM in unfractionated 61 CSF samples from 20 ALS patients and 20 HC individuals that were from our original cohort used in the discovery experiments and from an additional 10 ALS patients and 11 HC individuals that were from an independent cohort (Table 1). To accurately quantify the target peptides using PRM in CSF samples from ALS patients and HC individuals, SIL peptides were added to the CSF samples at the beginning of the experiment. We added similar concentrations of the SIL peptides to the endogenous peptides, but when the concentrations of the endogenous peptides were lower than 5 pmol/mL, we added 5 pmol/mL of SIL peptides to avoid ambiguous detection. After the digestion of the CSF proteins with trypsin, the endogenous and SIL peptides were monitored by PRM assay (Figure 3). In total, 56 peptides from 31 proteins were validated by PRM assay. The expression of APOB1 (two peptides), CHI3L1 (three peptides), CHIT1 (four peptides), GPNMB (one peptide), ERAP2 (one peptide), JCHAIN (one of two quantified peptides), SERPINA1 (three peptides), SERPINA3 (two peptides), and UCHL1 (one peptide) were upregulated in ALS patients (Figure 4A), while the expression of APP (three peptides), CAMK2A (three peptides), CLSTN3 (one peptide), FSTL4 (one of two quantified peptides), L1CAM (one peptide), and NPTX2 (two peptides) were downregulated (Figure 4B). The rest of the peptides did not show statistically significant differences (Figure S2). Interestingly, we did not find notable differences between the groups with ALSFRS scores ≥ 40 and the groups with ALSFRS scores < 39. To estimate the consistency of the discovery data with the validation data, we conducted a correlation analysis of the ALS candidate biomarker proteins that showed statistical significance in both the discovery and validation experiments. All the ALS candidate biomarkers showed a positive correlation between discovery and validation data (Figure 4C). We also conducted ROC analysis to evaluate the performance of 29 peptides with statistically significant differences in discriminating ALS from HC. CHIT1 (FTDMVATANNR with AUC 0.868, DNQWVGFDDVESFK with AUC 0.858, SSFYSCAAGR with AUC 0.869, and YPLIQTLR with AUC 0.843), APOB (LPYTIITTPPLK with AUC 0.837 and WNFYYSPQSSPDK with 0.792), CAMK2A (VTEQLIEAISNGDFESYTK with AUC 0.839, ITQYLDAGGIPR with AUC 0.829, and FYFENLWSR with AUC 0.789), and SERPINA3 (AVLDVFEEGTEASAATAVK with AUC 0.816 and LINDYVK with AUC 0.774) showed the top four highest average areas under the ROC curves among the measured candidate biomarkers (Figure 5 and Figure S3). The ROC analysis results for the remaining candidates are shown in Figure S3 and Table S2. To further improve the discriminability of the candidate biomarkers, we employed multivariate analysis using MCCV and PLS-DA methods. When all 56 features were used, the AUC was 0.906 (Figure 6A). CHIT1 (SSFYSCAAGR, FTDMVATANNR, and DNQWVGFDDVESFK) was the most contributing protein, followed by CAMK2A (VTEQLIEAISNGDFESYTK and ITQYLDAGGIPR) and APOB (LPYTIITTPPLK) (Figure 6B). The predictive accuracy reached 84% when 56 features were used (Figure 6C). These results showed that the proteins that we have identified hold promise as candidate biomarkers for ALS, and when multiple biomarkers are used, the discriminative power can be further improved.

## 4. Discussion

In this study, we used an unbiased quantitative discovery approach to identify proteins that are differentially present in ALS CSF compared with HC CSF and validated these differences using a targeted quantitative approach. Towards this end, we performed discovery-based studies on pre-fractionated CSF from 20 patients with ALS and 20 HC individuals using an 11-plex TMT platform and discovered 53 candidate ALS biomarker proteins (19 candidates with *q* < 0.05 and 34 candidates with *q* < 0.1). Among the 53 candidate ALS biomarker proteins, 44 proteins are novel, and 9 proteins were identified previously as being differentially expressed in ALS, and these include neurofilament proteins (NEFL, NEFM, and NEFH), CHIT1, CHI3L1, CHI3L2, APOB, GPNMB, and UCHL1 [20,41,42]. Neurofilament proteins are intermediate filament proteins that are elevated in response to neuronal damage [25,43], while CHIT1, CHI3L1, and CHI3L2 are members of the human chitinase family that are implicated in inflammation [24]. UCHL1 is a ubiquitin-protein hydrolase important for protein homeostasis that is expressed in neurons and neuroendocrine cells, whereas APOB is associated with metabolic changes in ALS [40,44]. Consistent with previous studies, we found that all these candidate proteins were elevated in CSF samples from patients with ALS [20,25,26,45,46,47,48]. This observation independently strengthens the potential of novel candidate biomarker for ALS identified in our study.

After the discovery phase, we utilized PRM analysis of unfractionated CSF without TMT labeling to validate the candidates that we had identified in the discovery phase of our study. Unfortunately, proteins that showed the largest differential expression in our discovery studies, such as neurofilament proteins, were not detectable by PRM, suggesting that these proteins are expressed at relatively lower levels in CSF. Since we used unfractionated CSF peptides in the validation phase while we used fractionated CSF peptides in the discovery phase, some candidate ALS biomarker proteins with low abundance were not detected, even though PRM analysis is more sensitive than TMT. Further validation of these candidates is needed to determine their utility as biomarkers for ALS.

Our PRM analyses successfully detected 31 proteins discovered from our discovery experiments. APOB, which was increased in ALS in our study, is a lipoprotein responsible for carrying fat molecules in the human body [49]. Mariosa et al. [40] reported that the higher APOB level in serum is associated with an increased ALS risk and may serve as prodromal symptoms decades before ALS diagnosis. Picard et al. [49] reported that APOB is a biomarker for early tau pathology in Alzheimer’s disease. APP, which was decreased in ALS in our study, has been proposed to be involved in several human neurodegenerative diseases such as AD, autism, fragile X syndrome (FXS), ALS, multiple sclerosis (MS), and Lesch–Nyhan disease (LND) [50]. Since β-amyloid accumulation in the brain of ALS patients has been reported [51], the β-amyloid accumulation in the brain of ALS can result in the decrease in β-amyloid in CSF. However, considering the APP peptides quantified in the PRM-MS analysis is not β-amyloid region, the decreased APP in ALS is less likely to be related to the β-amyloid accumulation. CAMK2A, which was decreased in ALS in our study, is a kinase crucial for neural plasticity and memory and is expressed in both excitatory and inhibitory neurons [51]. The expression of CAMK2A is decreased in inhibitory neurons at the initial stage of nerve injury [52]. Moreover, CAMK2A is reported to be dysregulated in the AD hippocampus [53]. CHI3L1 and CHIT1, which were increased in ALS in our study, are members of the human chitinase family proteins. Varghese et al. [26] reported that CSF levels of CHIT1, CHI3L1, and CHI3L2 were significantly elevated in ALS patients relative to HC individuals. CLSTN3, which was decreased in ALS in our study, is a calsyntenin member protein that plays an evolutionarily conserved role in memory and learning, promoting the development of synapses [54]. To the best of our knowledge, the current study is the first to characterize decreased CLSTN3 levels in human CSF from ALS patients. However, since studies on the involvement of CLSTN3 in ALS are still lacking, further mechanistic studies are needed. ERAP2, which was increased in ALS in our study, is a protein associated with immune-mediated diseases [55]. However, little is known about the relationship between ERAP2 and ALS. Ziff et al. [56] reported that human-induced pluripotent-stem-cell-derived astrocytes carrying ALS-causing mutations showed increased ERAP2 mRNA expressed with reduced intron retention (IR), which is required for the activation of astrocytes. The increased ERAP2 in ALS CSF could be potentially involved in the activation of astrocytes. FSTL4, which was decreased in ALS in our study, is known as a negative regulator of BDNF maturation [57]. Collins et al. [6] conducted mass-spectrometry-based biomarker discovery using ALS CSF and reported that FSTL4 was decreased in ALS CSF in consistency with our result. JCHAIN, which was increased in ALS in our study, is a protein component of immunoglobulins A and M working as a glue for the formation of multimeric immunoglobulins [58]. However, its relationship with ALS is not known yet. GPNMB, which was increased in ALS in our study, is a protein involved in inflammatory processes [59], and the absence of UCHL1 leads to the degeneration of motor axons [60]. Both GPNMB and UCHL1 have been reported as ALS candidate biomarkers recently, with increased expression levels in ALS consistent with our study [23]. L1CAM, which was decreased in ALS in our study, is involved in diverse processes at different stages during the development of the nervous system and is expressed on the surface of nerve cells [61,62]. Our data showing the decreased expression level of L1CAM in ALS CSF could be reflecting the death of neuronal cells. NPTX2, which was decreased in ALS in our study, is involved in mediating excitatory neurotransmission and has been reported to be involved in various diseases such as Parkinson’s disease (PD), ischemia, Alzheimer’s disease (AD), and frontotemporal dementia [63]. Xiao et al. [64] also reported that NPTX2 is involved in the adaptative control of the pyramidal neuron-fast-spiking parvalbumin circuit, and failure of this adaptive control could be involved in the cognitive decline of AD patients. The decreased NPTX2 in CSF in our data suggests that the adaptative control of the pyramidal neuron-fast-spiking parvalbumin circuit could be damaged in the ALS patient’s brain. SERPINA1, which was increased in ALS in our study, is an acute inflammatory protein, and the overexpression of this protein has been observed in AD and FTLD [65,66]. The increased level of SERPINA1 was also observed in Creutzfeldt–Jakob disease (CJD) and frontotemporal lobar degeneration (FTLD) [67]. The increased SERPINA1 level in our data suggests that the acute inflammatory response mediated by SERPINA1 could be involved in ALS as well. SERPINA3, which was increased in ALS in our study, is a glycoprotein involved in various physiological processes such as complement cascade, inflammation, and wound healing, and this protein is reported to be involved in various neurodegenerative diseases [68]. Fissolo et al. reported an increased level of SERPINA3 in CSF from progressive multiple sclerosis [69].

In this study, we were able to validate a substantial number of candidate ALS biomarker proteins identified in the discovery phase. However, there were still some proteins that could not be validated as they were not detectable in the PRM analysis, likely due to their abundance falling below the detection limit of the PRM method. Consequently, further studies will be necessary to validate these proteins.

## 5. Conclusions

Our data showed that the candidate biomarkers, such as CHIT1, APOB, CAMK2A, and SERPIN3, had AUC > 0.8 in the ROC curve analysis. Furthermore, the multivariate analysis using all the candidate biomarkers identified in this study showed even im-proved AUC > 0.9, suggesting that combining the ALS biomarker candidate proteins identified in our study with ALS biomarkers from other studies can potentially further improve the discriminatory power between ALS and control. In summary, our mass-spectrometry-based approach identified fifteen ALS candidate biomarker proteins. Further validation across a larger number of cohorts across ALS and other neurodegenerative diseases will be needed to evaluate their utility as ALS biomarkers.

## Figures and Tables

**Table 1:** The demographics of CSF samples used in this study.

Subject ID	Sample Type	Group	Gender	Race	Age	Application of the Samples in This Study
ALS01	CSF	ALSFRS 40	Male	White	62	Discovery and Validation
ALS02	CSF	ALSFRS 36	Female	White	68	Discovery and Validation
ALS03	CSF	ALSFRS 44	Male	White	54	Discovery and Validation
ALS04	CSF	ALSFRS 33	Female	White	55	Discovery and Validation
ALS05	CSF	ALSFRS 37	Female	White	44	Discovery and Validation
ALS06	CSF	ALSFRS 42	Female	White	51	Discovery and Validation
ALS07	CSF	ALSFRS 42	Male	White	64	Discovery and Validation
ALS08	CSF	ALSFRS 33	Female	White	52	Discovery and Validation
ALS09	CSF	ALSFRS 35	Male	White	68	Discovery and Validation
ALS10	CSF	ALSFRS 34	Male	White	55	Discovery and Validation
ALS11	CSF	ALSFRS 34	Male	White	61	Discovery and Validation
ALS12	CSF	ALSFRS 35	Male	White	79	Discovery and Validation
ALS13	CSF	ALSFRS 42	Male	White	63	Discovery and Validation
ALS14	CSF	ALSFRS 40	Male	White	59	Discovery and Validation
ALS15	CSF	ALSFRS 44	Female	White	66	Discovery and Validation
ALS16	CSF	ALSFRS 43	Male	White	73	Discovery and Validation
ALS17	CSF	ALSFRS 41	Male	White	55	Discovery and Validation
ALS18	CSF	ALSFRS 48	Male	White	65	Discovery and Validation
ALS19	CSF	ALSFRS 46	Male	White	40	Discovery and Validation
ALS20	CSF	ALSFRS 34	Female	White	53	Discovery and Validation
ALS21	CSF	No score	Male	White	64	Validation
ALS22	CSF	ALSFRS 32	Male	White	58	Validation
ALS23	CSF	ALSFRS 42	Male	White	45	Validation
ALS24	CSF	ALSFRS 40	Female	White	41	Validation
ALS25	CSF	ALSFRS 47	Male	White	44	Validation
ALS26	CSF	ALSFRS 41	Male	White	51	Validation
ALS27	CSF	ALSFRS 40	Male	White	68	Validation
ALS28	CSF	ALSFRS 27	Female	White	44	Validation
ALS29	CSF	ALSFRS 48	Male	White	57	Validation
ALS30	CSF	ALSFRS 15	Female	White	63	Validation
HC01	CSF	Healthy Control	Male	White	38	Discovery and Validation
HC02	CSF	Healthy Control	Male	White	58	Discovery and Validation
HC03	CSF	Healthy Control	Female	White	23	Discovery and Validation
HC04	CSF	Healthy Control	Female	White	58	Discovery and Validation
HC05	CSF	Healthy Control	Female	Asian	43	Discovery and Validation
HC06	CSF	Healthy Control	Male	White	35	Discovery and Validation
HC07	CSF	Healthy Control	Male	White	57	Discovery and Validation
HC08	CSF	Healthy Control	Female	White	46	Discovery and Validation
HC09	CSF	Healthy Control	Male	White	76	Discovery and Validation
HC10	CSF	Healthy Control	Female	White	44	Discovery and Validation
HC11	CSF	Healthy Control	Female	White	47	Discovery and Validation
HC12	CSF	Healthy Control	Male	White	70	Discovery and Validation
HC13	CSF	Healthy Control	Female	White	62	Discovery and Validation
HC14	CSF	Healthy Control	Male	White	74	Discovery and Validation
HC15	CSF	Healthy Control	Male	White	66	Discovery and Validation
HC16	CSF	Healthy Control	Male	White	72	Discovery and Validation
HC17	CSF	Healthy Control	Female	White	62	Discovery and Validation
HC18	CSF	Healthy Control	Female	White	66	Discovery and Validation
HC19	CSF	Healthy Control	Male	White	69	Discovery and Validation
HC20	CSF	Healthy Control	Female	White	55	Discovery and Validation
HC21	CSF	Healthy Control	Female	White	61	Validation
HC22	CSF	Healthy Control	Male	White	66	Validation
HC23	CSF	Healthy Control	Male	White	67	Validation
HC24	CSF	Healthy Control	Female	White	64	Validation
HC25	CSF	Healthy Control	Female	ND	59	Validation
HC26	CSF	Healthy Control	Male	White	65	Validation
HC27	CSF	Healthy Control	Male	White	66	Validation
HC28	CSF	Healthy Control	Female	White	58	Validation
HC29	CSF	Healthy Control	Male	White	54	Validation
HC30	CSF	Healthy Control	Female	White	59	Validation
HC31	CSF	Healthy Control	Female	ND	60	Validation

HC: healthy control; ALS: amyotrophic lateral sclerosis; ALSFRS: Amyotrophic Lateral Sclerosis Functional Rating Scale.

**Table 2:** Differentially expressed proteins in CSFs from ALS patients compared to the ones from HC individuals identified in the discovery experiment.

Name	Gene Symbol	*p* Value	*q*-Value	Log_2_ (ALS/HC)	Detectability by PRM	*q*-Value
Chitinase-3-like protein 2	*CHI3L2*	2.01 × 10^−8^	0	2.527697		<0.05
Chitotriosidase-1	*CHIT1*	1.88 × 10^−5^	0	3.99428	Detectable	
Neurofilament light polypeptide	*NEFL*	1.47 × 10^−7^	0	1.722488		
Neurofilament medium polypeptide	*NEFM*	6.97 × 10^−11^	0	3.784436		
Neurofilament heavy polypeptide	*NEFH*	5.91 × 10^−5^	0.001333	2.779995		
Alpha-1-antichymotrypsin	*SERPINA3*	6.94 × 10^−6^	0.008571	1.077944	Detectable	
Ubiquitin carboxyl-terminal hydrolase isozyme L1	*UCHL1*	1.48 × 10^−5^	0.015	1.012955	Detectable	
Transmembrane glycoprotein NMB	*GPNMB*	8.06 × 10^−5^	0.0244	1.069444	Detectable	
Multiple epidermal-growth-factor-like domains protein 8	*MEGF8*	4.36 × 10^−6^	0.026182	−0.72796	Detectable	
Aspartate aminotransferase, mitochondrial	*GOT2*	0.00032	0.026222	1.511867		
Centromere protein F	*CENPF*	0.001221	0.028	2.205369		
Neuronal pentraxin-2	*NPTX2*	0.00016	0.028667	−1.13379	Detectable	
Calcium/calmodulin-dependent protein kinase type II subunit alpha	*CAMK2A*	0.000116	0.033143	−0.95518	Detectable	
Apolipoprotein B-100	*APOB*	0.002532	0.041053	2.13572	Detectable	
Amyloid-beta A4 protein	*APP*	6.85 × 10^−5^	0.042444	−0.75982	Detectable	
Neural cell adhesion molecule L1	*L1CAM*	7.59 × 10^−5^	0.043	−0.74116	Detectable	
Chitinase-3-like protein 1	*CHI3L1*	0.000583	0.044	1.137858	Detectable	
Platelet factor 4	*PF4*	0.002747	0.044533	2.573066		
Lysozyme C	*LYZ*	0.000368	0.0465	1.015916		
Calsyntenin-1	*CLSTN1*	0.000146	0.051429	−0.75724	Detectable	<0.1
Contactin-associated protein-like 2	*CNTNAP2*	0.000225	0.052182	−0.79877	Detectable	
Macrophage-capping protein	*CAPG*	0.00037	0.056522	0.840849		
Triosephosphate isomerase	*TPI1*	2.87 × 10^−5^	0.05712	−0.59761	Detectable	
Microfibril-associated glycoprotein 4	*MFAP4*	0.00012	0.058429	−0.66703	Detectable	
Endoplasmic reticulum aminopeptidase 2	*ERAP2*	0.001754	0.058897	1.147899	Detectable	
Gamma-crystallin D	*CRYGD*	0.007001	0.058963	−2.86543		
Syndecan binding protein (Syntenin), isoform CRA_a	*SDCBP*	0.000461	0.059	0.846597		
Follistatin-related protein 4	*FSTL4*	0.000542	0.059333	−0.82485	Detectable	
Transport and Golgi organization protein 1 homolog	*MIA3*	4.14 × 10^−7^	0.061231	−0.45853		
Neurocan core protein	*NCAN*	0.000476	0.070545	−0.73593	Detectable	
Platelet basic protein	*PPBP*	0.006893	0.072625	2.127046	Detectable	
Leukotriene A-4 hydrolase	*LTA4H*	8.72 × 10^−5^	0.073677	0.593814	Detectable	
Chondroitin sulfate proteoglycan 5	*CSPG5*	0.001286	0.083412	−0.84017	Detectable	
Band 4.1-like protein 3	*EPB41L3*	0.004408	0.092686	1.101059		
Immunoglobulin heavy variable 2-5	*IGHV2-5*	0.010187	0.096522	1.802516		
Plectin	*PLEC*	0.00037	0.096681	0.586559		
Myosin light chain kinase, smooth muscle	*MYLK*	0.000406	0.09688	0.572703		
Calsyntenin-3	*CLSTN3*	8.53 × 10^−5^	0.097255	−0.49128	Detectable	
Serine/threonine-protein phosphatase 2A activator	*PTPA*	0.000505	0.0978	0.637236		
UMP-CMP kinase	*CMPK1*	0.002623	0.097956	0.843523		
Neuronal pentraxin-1	*NPTX1*	0.002511	0.098222	−0.79414	Detectable	
Alpha-1-antitrypsin	*SERPINA1*	0.002667	0.098449	0.842153	Detectable	
Leucine-rich repeat and immunoglobulin-like domain-containing nogo receptor-interacting protein 1	*LINGO1*	0.000955	0.098462	−0.65034	Detectable	
Immunoglobulin lambda variable 3-19	*IGLV3-19*	0.006846	0.098537	1.408966	Detectable	
Immunoglobulin lambda variable 3-27	*IGLV3-27*	0.003614	0.098566	0.886375		
Alpha-2,8-sialyltransferase 8E	*ST8SIA5*	0.000228	0.098974	−0.5754	Detectable	
Butyrophilin subfamily 3 member A2	*BTN3A2*	0.005124	0.099	1.036734		
Reticulon-4 receptor-like 2	*RTN4RL2*	0.00064	0.099111	−0.67662	Detectable	
Protein kinase C-binding protein NELL2	*NELL2*	0.001377	0.099135	−0.78332	Detectable	
Immunoglobulin J chain	*JCHAIN*	0.010018	0.099167	1.661328	Detectable	
Multiple epidermal-growth-factor-like domains protein 9	*MEGF9*	0.000603	0.099263	−0.66189		
Glutathione S-transferase Mu 1	*GSTM1*	0.008847	0.09981	1.620058		
Neurofascin	*NFASC*	0.00052	0.099907	−0.62704	Detectable	

## Data Availability

All mass spectrometry data and search results have been deposited in the ProteomeXchange Consortium via the PRIDE partner repository with the dataset identifierPXD038846 and project name ‘Discovery of biomarkers for amyotrophic lateral sclerosis from human cerebrospinal fluid using mass spectrometry-based proteomics’ [70].

## Supplementary Materials

The following supporting information can be downloaded at: https://www.mdpi.com/article/10.3390/biomedicines11051250/s1, Figure S1: The response curve of candidate biomarker peptides in the PRM analysis; Figure S2: The peptides that did not show statistically different differences between ALS and HC in the PRM-MS assay; Figure S3: ROC analyses of peptides from the candidate ALS biomarker proteins from the discovery experiment; Table S1: The list of peptides detected in the PRM-MS analysis; Table S2: Average abundance of each peptide quantified by PRM-MS and their statistical analysis results. Supplementary Data S1: The list of identified proteins by the TMT-based proteomics experiment.

## Abbreviations

ACN, acetonitrile; AGC, automatic gain control; AUC, area under the curve; ALS, amyotrophic lateral sclerosis; ALSFRS, Amyotrophic Lateral Sclerosis Functional Rating Scale; bRPLC, basic pH reversed-phase liquid chromatography; CAA, chloroacetamide; CSF, cerebrospinal fluids; CV, coefficient variation; DDA, data-dependent acquisition; FA, formic acid; FDR, false-discovery rate; HC, healthy control; MS, mass spectrometry; MS/MS, fragment ion; MS1, precursor ion; MCCV, Monte Carlo cross-validation; MP, master pool; PLS-DA, partial least squares discriminant analysis; PSM, peptide-spectrum match; PRM, parallel reaction monitoring; ROC, receiver operating characteristic; RT, room temperature; SAM, significance analysis of microarrays; SIL, standard isotope labeled; TEAB, triethylammonium bicarbonate; TECP, tris (2-carboxyethyl) phosphine hydrochloride; TFA, trifluoroacetic acid; TMT, tandem mass tag.
