# Supplementary material for: Discovery of Biomarkers for Amyotrophic Lateral Sclerosis from Human Cerebrospinal Fluid Using Mass-Spectrometry-Based Proteomics
Source: Biomedicines. 2023 Apr 23;11(5):1250. doi: 10.3390/biomedicines11051250 (PMC10215566; doi:10.3390/biomedicines11051250)
Supplement: Supplementary file 1 [file biomedicines-11-01250-s001.zip › biomedicines-11-01250+supplementary.pdf]

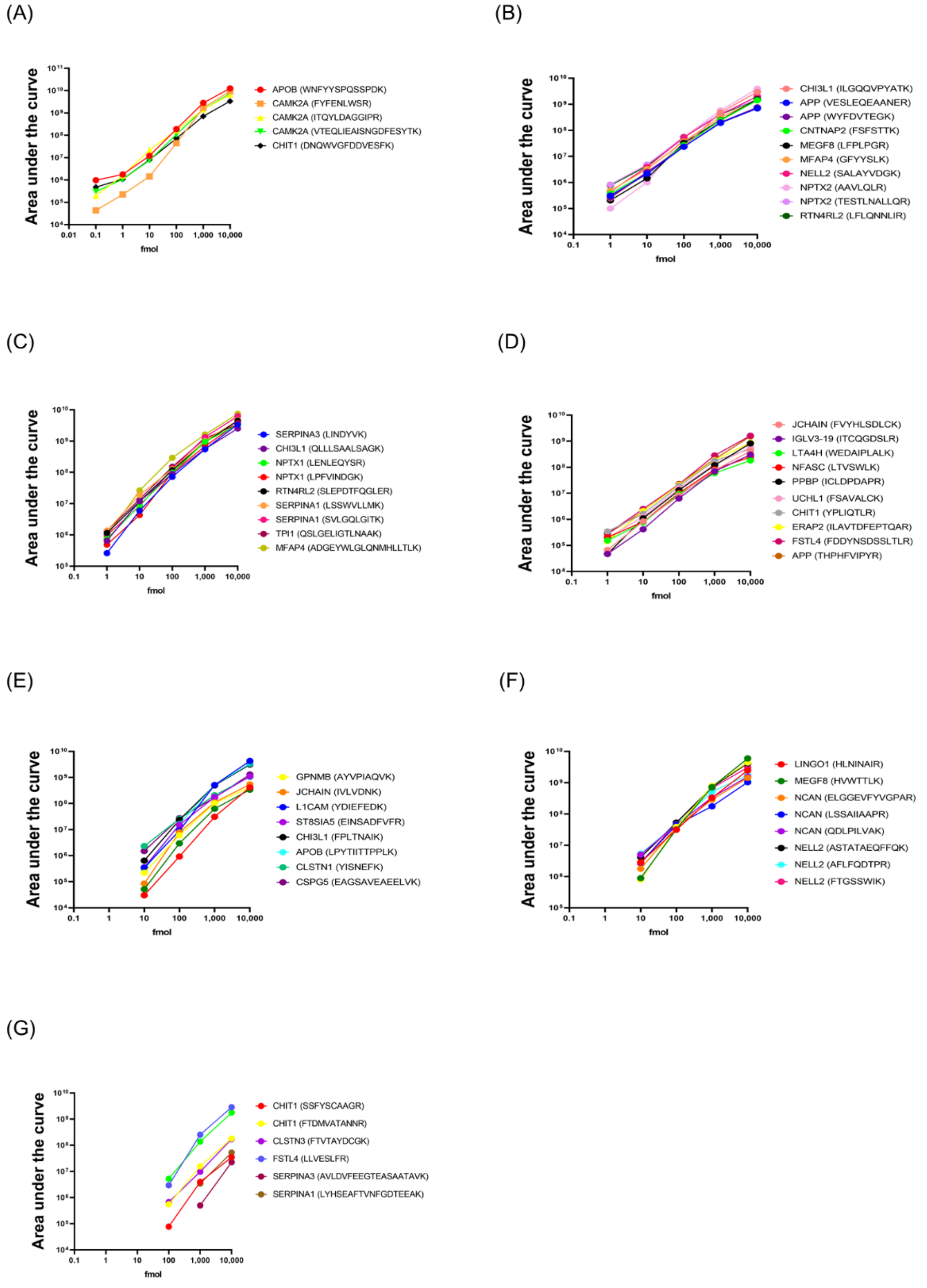

Supplemental Figure S1. The response curve of candidate biomarker peptides in the PRM analysis.

The SIL standard peptides ranging from 0.01 fmol to 10 pmol were added to the CSF samples and quantified by the PRM analysis to monitor the dynamic range of each target peptide. The peptides with various response curves are shown; 0.01 to 10,000 fmol (A), 0.1 to 10,000 fmol (B, C, and D), 1 to 10,000 fmol (E and F), and 10 or 100 fmol to 10,000 mol (G).

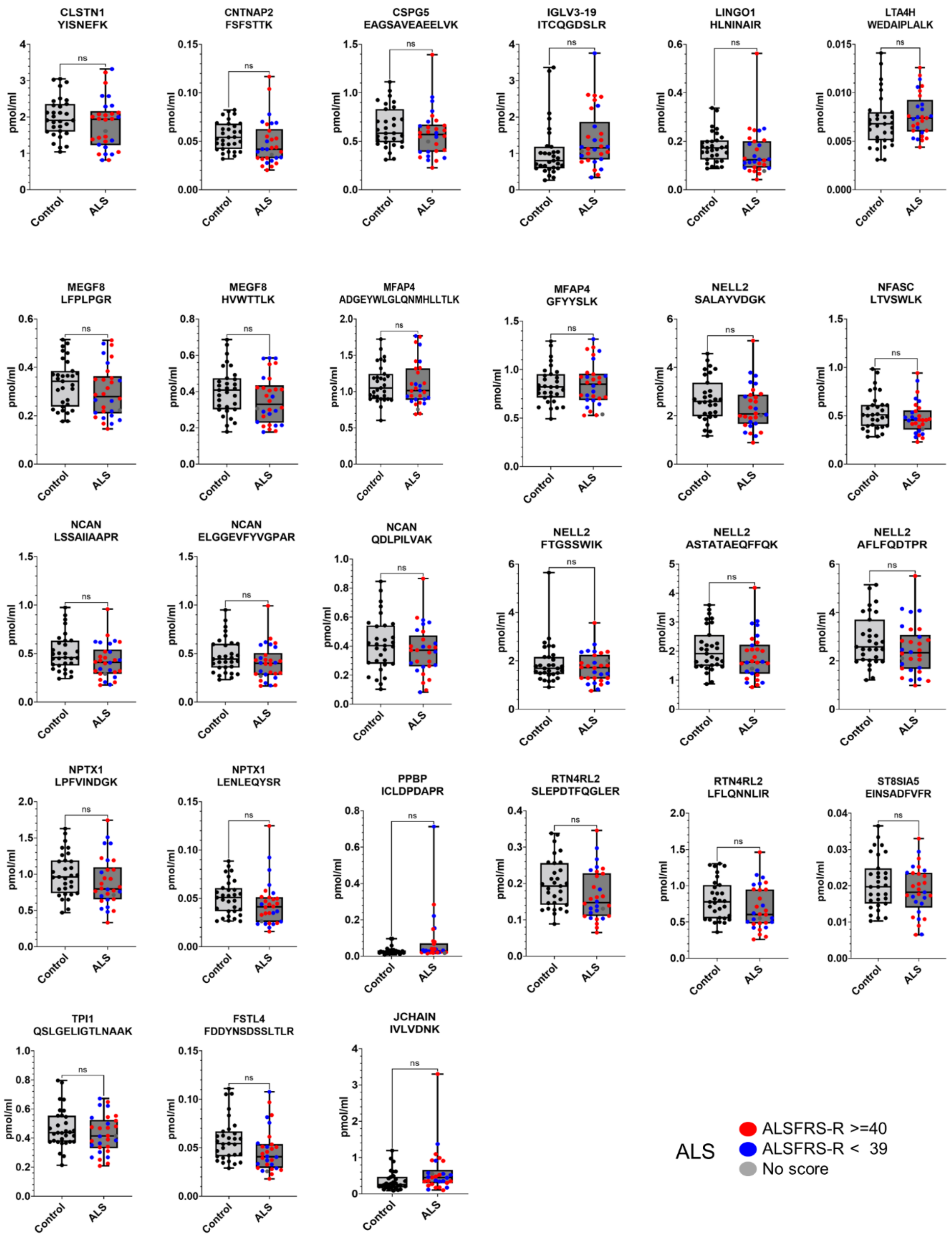

Supplemental Figure S2. The peptides that did not show statistically different differences between ALS and HC in the PRM-MS assay.

The peptides of the ALS candidate biomarker proteins identified in the discovery experiment were validated by PRM-MS. The peptides that did not show statistically significant differences between ALS and HC in the PRM-MS analyses are shown here. Patients with ALSFRS scores < 39 were shown in blue dots, and patients with ALSFRS scores ≥ 40 were shown in red dots. Since one ALS patient had no score, the patient was shown in a gray dot. All PRM-MS analyses were performed in 3 technical replicates. Student's unpaired, two-tailed *t*-test was conducted for statistical analysis between groups (ns: not significant).

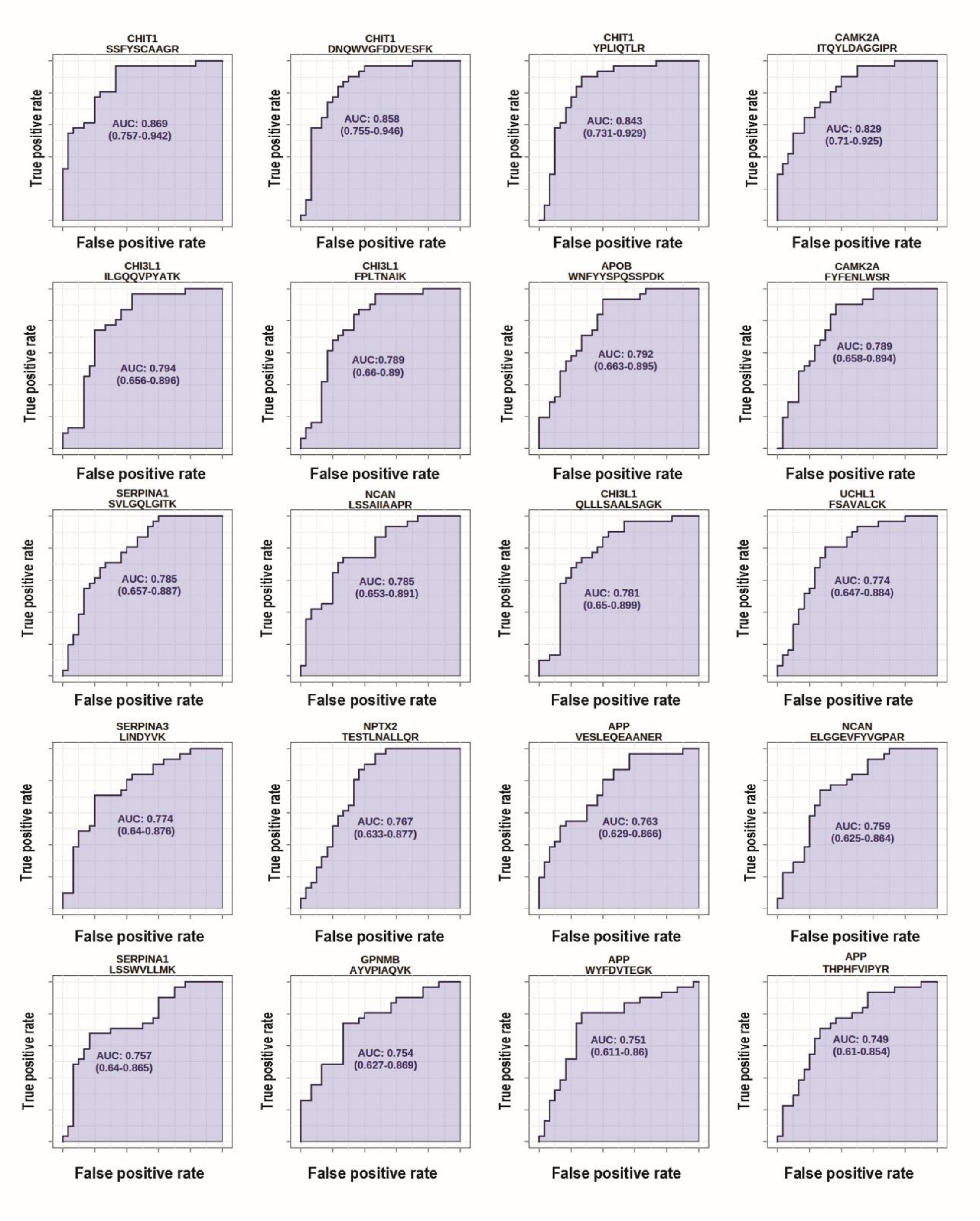
CHIT1 SSFYSCAAGR
AUC: 0.869 (0.757-0.942)
CHIT1 DNQWVGFDDVESFK
AUC: 0.858 (0.755-0.946)
CHIT1 YPLIQTLR
AUC: 0.843 (0.731-0.929)
CAMK2A ITQYLDAGGIPR
AUC: 0.829 (0.71-0.925)
CHI3L1 ILGQQVPYATK
AUC: 0.794 (0.656-0.896)
CHI3L1 FPLTNAIK
AUC:0.789 (0.66-0.89)
APOB WNFYYSPQSSPDK
AUC: 0.792 (0.663-0.895)
CAMK2A FYFENLWSR
AUC: 0.789 (0.658-0.894)
SERPINA1 SVLGQLGITK
AUC: 0.785 (0.657-0.887)
NCAN LSSAIIAAPR
AUC: 0.785 (0.653-0.891)
CHI3L1 QLLLSAALSAGK
AUC: 0.781 (0.65-0.899)
UCHL1 FSAVALCK
AUC: 0.774 (0.647-0.884)
SERPINA3 LINDYVK
AUC: 0.774 (0.64-0.876)
NPTX2 TESTLNALLQR
AUC: 0.767 (0.633-0.877)
APP VESLEQEAANER
AUC: 0.763 (0.629-0.866)
NCAN ELGGEVFYVGPAR
AUC: 0.759 (0.625-0.864)
SERPINA1 LSSWVLLMK
AUC: 0.757 (0.64-0.865)
GPNMB AYVPIAQVK
AUC: 0.754 (0.627-0.869)
APP WYFDVTEGK
AUC: 0.751 (0.611-0.86)
APP THPHFVIPYR
AUC: 0.749 (0.61-0.854)
True positive rate
False positive rate

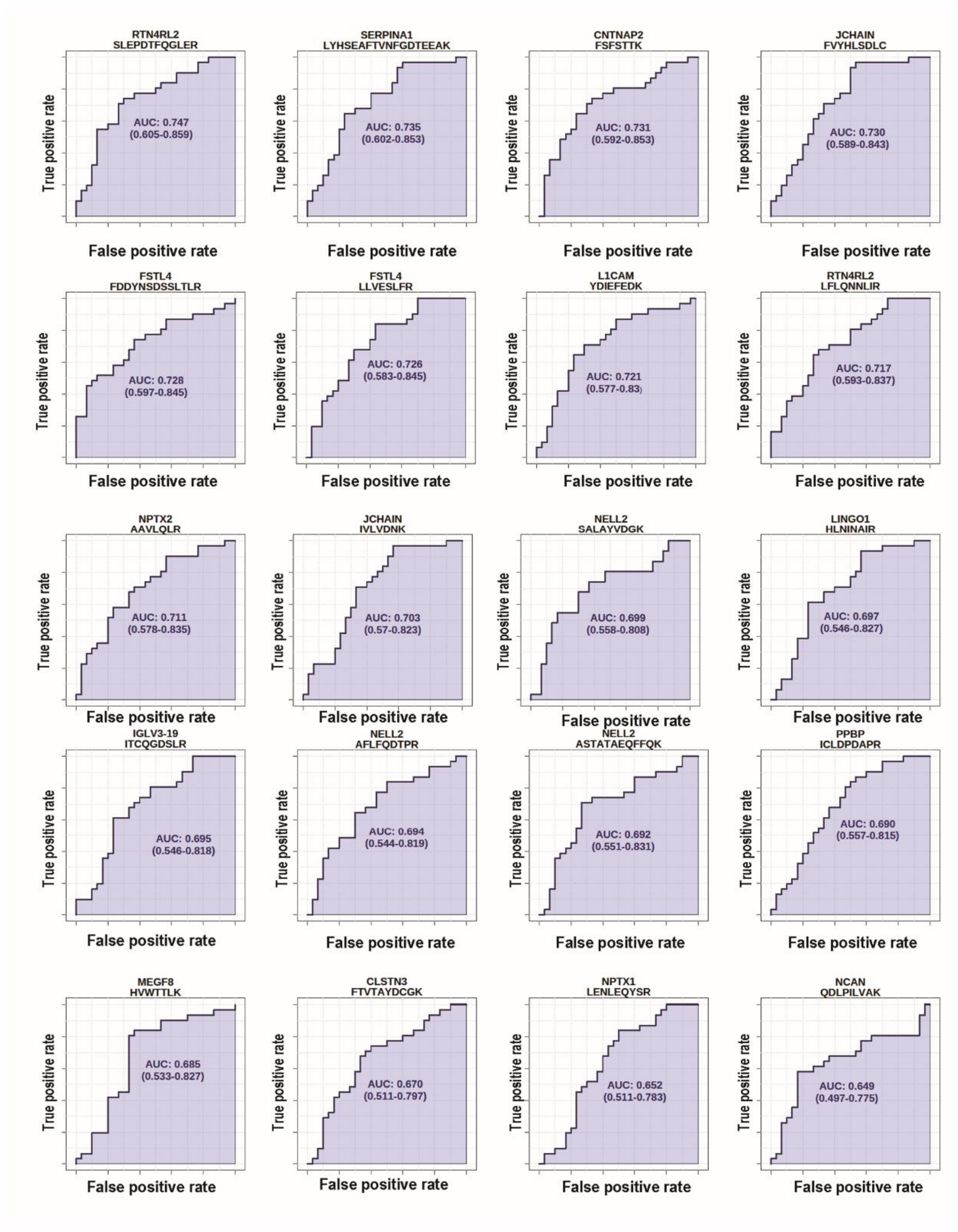
RTN4RL2
SLEPDTFQGLER
AUC: 0.747
(0.605-0.859)
SERPINA1
LYHSEAFTVNFGDTEEAK
AUC: 0.735
(0.602-0.853)
CNTNAP2
FSFSTTK
AUC: 0.731
(0.592-0.853)
JCHAIN
FVYHLSDLC
AUC: 0.730
(0.589-0.843)
FSTL4
FDDYNSDSSLTLR
AUC: 0.728
(0.597-0.845)
FSTL4
LLVESLFR
AUC: 0.726
(0.583-0.845)
L1CAM
YDIEFEDK
AUC: 0.721
(0.577-0.83)
RTN4RL2
LFLQNNLIR
AUC: 0.717
(0.593-0.837)
NPTX2
AAVLQLR
AUC: 0.711
(0.578-0.835)
JCHAIN
IVLVDNK
AUC: 0.703
(0.57-0.823)
NELL2
SALAYVDGK
AUC: 0.699
(0.558-0.808)
LINGO1
HLNINAIR
AUC: 0.697
(0.546-0.827)
IGLV3-19
ITCQGDSLR
AUC: 0.695
(0.546-0.818)
NELL2
AFLFQDTPR
AUC: 0.694
(0.544-0.819)
NELL2
ASTATAEQFFQK
AUC: 0.692
(0.551-0.831)
PPBP
ICLDPDAPR
AUC: 0.690
(0.557-0.815)
MEGF8
HVWTTLK
AUC: 0.685
(0.533-0.827)
CLSTN3
FTVTAYDCGK
AUC: 0.670
(0.511-0.797)
NPTX1
LENLEQYSR
AUC: 0.652
(0.511-0.783)
NCAN
QDLPILVAK
AUC: 0.649
(0.497-0.775)
True positive rate
False positive rate

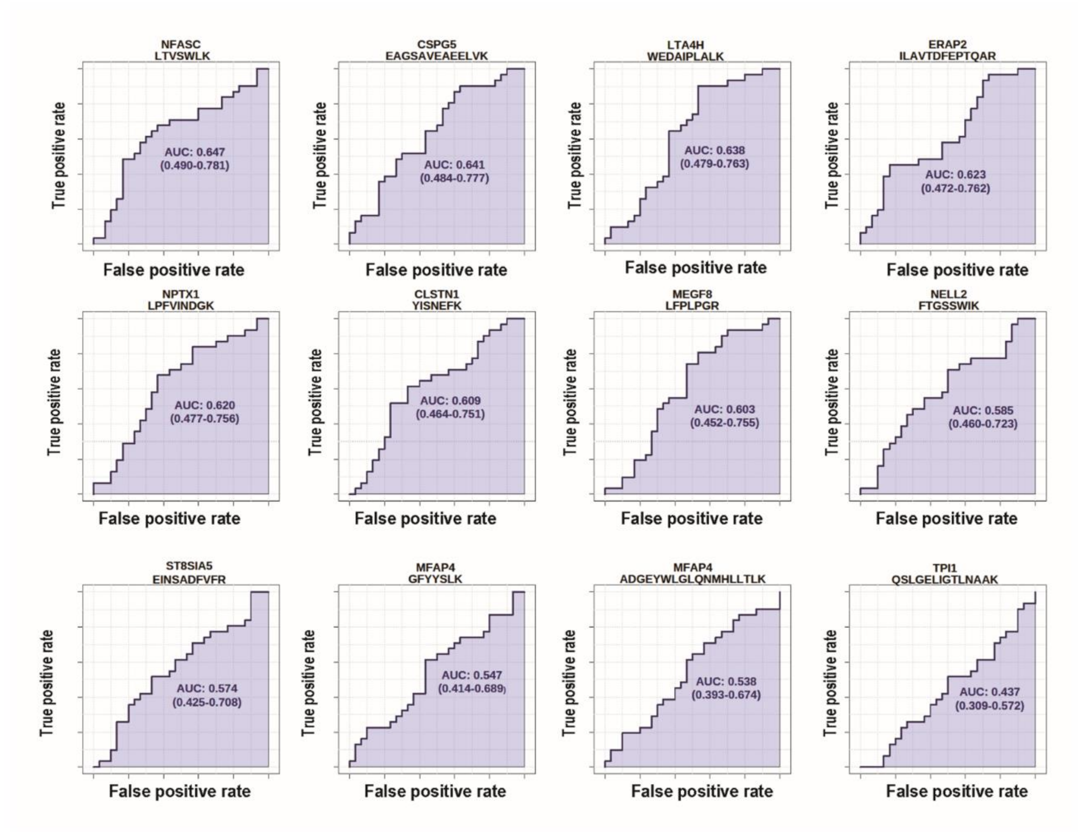

Supplemental Table S3. ROC analyses of peptides from the candidate ALS biomarker proteins from the discovery experiment.

ROC analysis was generated by bootstrapping. The values in the parenthesis show the lower and upper AUC values of 95% confidence interval. X-axis denotes a false positive rate (1-specificity) and Y-axis denotes a true positive rate (sensitivity).

Supplemental Table S1. The list of peptides detected in the PRM-MS analysis.

Gene symbol	Peptide sequence	light (m/z)	heavy (m/z)	Charge state (+)	HCD fragmentation energy	Maximum ion injection time
APOB	LPYTIITTPPLK	678.9105	682.9176	2	32	50
APOB	WNFYYSPQSSPDK	809.8623	813.8694	2	24	50
APP	VESLEQEAANER	687.8284	692.8326	2	32	100
APP	THPHFVIPYR	633.8407	638.8449	2	32	100
APP	WYFDVTEGK	572.7691	576.7762	2	32	100
CAMK2A	VTEQLIEAISNGDFESYTK	1072.523	1076.53	2	24	50
CAMK2A	FYFENLWSR	631.3037	636.3078	2	20	50
CAMK2A	ITQYLDAGGIPR	652.3539	657.358	2	20	50
CHI3L1	QLLLSAALSAGK	586.3559	590.363	2	32	100
CHI3L1	FPLTNAIK	452.2686	456.2757	2	32	100
CHI3L1	ILGQQVPYATK	609.3481	613.3552	2	32	100
CHIT1	YPLIQTLR	502.3004	507.3045	2	32	50
CHIT1	DNQWVGFDDVESFK	843.3757	847.3828	2	20	50
CHIT1	SSFYSCAAGR	553.2402	558.2443	2	20	50
CHIT1	FTDMVATANNR	620.293	625.2971	2	20	50
CLSTN1	YISNEFK	450.7267	454.7338	2	32	100
CLSTN3	FTVTAYDCGK	581.2659	585.273	2	20	50
CNTNAP2	FSFSTTK	409.2082	413.2153	2	32	100
CSPG5	EAGSAVEAEELVK	666.3381	670.3452	2	32	100
ERAP2	ILAVTDFEPTQAR	730.8908	735.895	2	24	50
FSTL4	FDDYNSDSSLTLR	766.8468	771.8509	2	24	50
FSTL4	LLVESLFR	488.7949	493.7991	2	24	50
GPNMB	AYVPIAQVK	494.7949	498.802	2	28	50
IGLV3-19	ITCQGDSLR	525.2558	530.26	2	28	50
JCHAIN	IVLVDNK	400.7475	404.7546	2	20	50
JCHAIN	FVYHLSDLCK	641.3184	645.3255	2	20	50
L1CAM	YDIEFEDK	529.7375	533.7446	2	24	50
LINGO1	HLNINAIR	475.7802	480.7843	2	32	100
LTA4H	WEDAIPLALK	578.3241	582.3312	2	24	50
MEGF8	HVWTTLK	442.7531	446.7602	2	32	100
MEGF8	LFPLPGR	400.2449	405.249	2	32	100
MFAP4	GFYYSLK	439.2264	443.2335	2	32	100
MFAP4	ADGEYWLGLQNMHLLTLK	701.3628	704.0342	3	32	100
NCAN	ELGGEVFYVGPAR	697.3592	702.3633	2	32	100
NCAN	LSSAIIAAPR	499.8033	504.8074	2	32	100
NCAN	QDLPILVAK	498.808	502.8151	2	32	100
NELL2	FTGSSWIK	463.2425	467.2496	2	32	100
NELL2	SALAYVDGK	462.2453	466.2524	2	32	100
NELL2	AFLFQDTPR	547.7851	552.7892	2	32	100
NELL2	ASTATAEQFFQK	664.8277	668.8348	2	32	100
NFASC	LTVSWLK	423.7578	427.7649	2	24	50
NPTX1	LPFVINDGK	501.7846	505.7917	2	32	100
NPTX1	LENLEQYSR	576.2882	581.2923	2	32	100
NPTX2	AAVLQLR	385.7478	390.7519	2	32	100
NPTX2	TESTLNALLQR	623.3435	628.3476	2	32	100
PPBP	ICLDPDAPR	528.7608	533.7649	2	20	50
RTN4RL2	LFLQNNLIR	565.8377	570.8418	2	32	100
RTN4RL2	SLEPDTFQGLER	696.3437	701.3478	2	32	100
SERPINA1	SVLGQLGITK	508.3109	512.318	2	32	100
SERPINA1	LYHSEAFTVNFGDTEEAK	686.6532	689.3246	3	32	100
SERPINA1	LSSWVLLMK	538.8123	542.8194	2	32	100
SERPINA3	AVLDVFEEGTEASAATAVK	636.6581	639.3295	3	32	100
SERPINA3	LINDYVK	432.7449	436.752	2	32	100
ST8SIA5	EINSADFVFR	599.2986	604.3027	2	24	50
TPI1	QSLGELIGTLNAAK	707.8986	711.9057	2	32	100
UCHL1	FSAVALCK	448.2389	452.246	2	24	50

Supplemental Table S2. Average abundance of each peptide quantified by PRM-MS and their statistical analysis results.

Name	Peptide sequence	Control (pmol/ml)	ALS (pmol/ml)	*P* value	Area under ROC curve
APOB	LPYTIITTPPLK	0.008	0.029	0.015	0.837
APOB	WNFYYSPQSSPDK	0.039	0.122	0.017	0.792
APP	VESLEQEAANER	4.37	3.459	0.008	0.763
APP	THPHFVIPYR	13.10	11.00	0.035	0.749
APP	WYFDVTEGK	2.86	2.386	0.037	0.751
CAMK2A	VTEQLIEAISNGDFESYTK	2.498	1.865	0.01	0.839
CAMK2A	ITQYLDAGGIPR	0.535	0.412	0.014	0.829
CAMK2A	FYFENLWSR	0.402	0.316	0.03	0.789
CHI3L1	ILGQQVPYATK	0.184	0.261	0.004	0.794
CHI3L1	QLLLSAALSAGK	0.977	1.359	0.006	0.781
CHI3L1	FPLTNAIK	0.229	0.317	0.009	0.789
CHIT1	FTDMVATANNR	0.041	0.365	9E-04	0.868
CHIT1	DNQWVGFDDVESFK	0.041	0.356	0.001	0.858
CHIT1	SSFYSCAAGR	0.042	0.338	0.002	0.869
CHIT1	YPLIQTLR	0.011	0.084	0.011	0.843
CLSTN1	YISNEFK	1.999	1.801	0.219	0.609
CLSTN3	FTVTAYDCGK	0.031	0.025	0.002	0.670
CNTNAP2	FSFSTTK	0.056	0.048	0.144	0.731
CSPG5	EAGSAVEAEELVK	0.643	0.576	0.247	0.641
ERAP2	ILAVTDFEPTQAR	0.006	0.009	0.044	0.623
FSTL4	LLVESLFR	0.051	0.039	0.008	0.726
FSTL4	FDDYNSDSSLTLR	0.058	0.047	0.055	0.728
GPNMB	AYVPIAQVK	0.098	0.127	0.02	0.754
IGLV3-19	ITCQGDSLR	1.05	1.395	0.087	0.695
JCHAIN	FVYHLSDLCK	0.337	0.57	0.037	0.730
JCHAIN	IVLVDNK	0.36	0.591	0.055	0.703
L1CAM	YDIEFEDK	0.099	0.083	0.034	0.721
LINGO1	HLNINAIR	0.176	0.154	0.286	0.697
LTA4H	WEDAIPLALK	0.007	0.008	0.305	0.638
MEGF8	HVWTTLK	0.405	0.355	0.138	0.685
MEGF8	LFPLPGR	0.332	0.3	0.231	0.603
MFAP4	GFYYSLK	0.838	0.849	0.837	0.547
MFAP4	ADGEYWLGLQNMHLLTLK	1.109	1.103	0.934	0.538
NCAN	LSSAIIAAPR	0.511	0.422	0.065	0.785
NCAN	ELGGEVFYVGPAR	0.481	0.404	0.103	0.759
NCAN	QDLPILVAK	0.413	0.368	0.338	0.649
NELL2	SALAYVDGK	2.694	2.335	0.129	0.699
NELL2	AFLFQDTPR	2.852	2.478	0.169	0.694
NELL2	ASTATAEQFFQK	2.064	1.803	0.186	0.692
NELL2	FTGSSWIK	1.910	1.721	0.332	0.585
NFASC	LTVSWLK	0.541	0.481	0.19	0.647
NPTX1	LENLEQYSR	0.051	0.044	0.206	0.652
NPTX1	LPFVINDGK	0.995	0.892	0.212	0.620
NPTX2	TESTLNALLQR	0.226	0.171	0.005	0.767
NPTX2	AAVLQLR	0.255	0.202	0.031	0.711
PPBP	ICLDPDAPR	0.029	0.078	0.054	0.690
RTN4RL2	SLEPDTFQGLER	0.199	0.167	0.077	0.747
RTN4RL2	LFLQNNLIR	0.807	0.688	0.108	0.717
SERPINA1	SVLGQLGITK	81.54	110.2	0.002	0.785
SERPINA1	LSSWVLLMK	71.00	89.51	0.021	0.757
SERPINA1	LYHSEAFTVNFGDTEEAK	80.87	94.96	0.036	0.735
SERPINA3	AVLDVFEEGTEASAATAVK	56.08	75.85	0.0004	0.816

SERPINA3	LINDYVK	1.329	1.692	0.011	0.774
ST8SIA5	EINSADFVFR	0.021	0.019	0.247	0.574
TPI1	QSLGELIGTLNAAK	0.467	0.425	0.221	0.437
UCHL1	FSAVALCK	0.008	0.01	0.0007	0.774
